# Molecular detection of *Porcine circovirus type 2* in swine herds of Eastern Cape Province South Africa

**DOI:** 10.1186/s12866-017-1121-4

**Published:** 2017-11-02

**Authors:** Kayode Olayinka Afolabi, Benson Chuks Iweriebor, Larry Chikwelu Obi, Anthony Ifeanyi Okoh

**Affiliations:** 1 Applied and Environmental Microbiology Research Group (AEMREG), Department of Biochemistry and Microbiology, University of Fort Hare, Private Bag X1314, Alice, Eastern Cape Province 5700 South Africa; 2 SAMRC Microbial Water Quality Monitoring Centre, University of Fort Hare, Private Bag X1314, Alice, Eastern Cape Province 5700 South Africa; 3 Academic and Research Division, University of Fort Hare, Private Bag X1314, Alice, Eastern Cape Province South Africa

**Keywords:** Porcine circovirus type 2, Eastern Cape Province, South Africa

## Abstract

**Background:**

*Porcine circovirus type 2* (PCV2) remains the main causative viral pathogen of porcine circovirus-associated diseases (PCVAD) of great economic importance in pig industry globally. This present study aims at determining the occurrence of the viral pathogen in swine herds of the Province.

**Results:**

The data obtained revealed that 15.93% of the screened samples (54/339) from the swine herds of the studied areas were positive for PCV2; while the severity of occurrence of the viral pathogen as observed at farm level ranges from approximately 5.6 to 60% in the studied farms. The majority (15 out of 17 = 88%) of the analyzed sequences were found clustering with other PCV2b strains in the phylogenetic analysis. More interestingly, two other sequences obtained were also found clustering within PCV2d genogroup, which is presently another fast-spreading genotype with observable higher virulence in global swine herds.

**Conclusion:**

This is the first report of PCV2 in swine herds of the Province and the first detection of PCV2b and PCV2d in South African swine herds. It follows the first reported case of PCV2a in an outbreak of porcine multisystemic wasting syndrome (PMWS) in Gauteng Province, South Africa more than one decade ago. This finding confirmed the presence of this all-important viral pathogen in pigs of the region; which could result in a serious outbreak of PCVAD and huge economic loss at the instances of triggering factors if no appropriate measures are taken to effectively curb its spread.

**Electronic supplementary material:**

The online version of this article (10.1186/s12866-017-1121-4) contains supplementary material, which is available to authorized users.

## Background

Porcine circoviruses (PCVs) are of the genus *Circovirus* in the family Circoviridae. They are non-enveloped viruses with a single-stranded circular DNA genome [1]. They are the smallest known animal viruses and include porcine circovirus type 1 (PCV1) and porcine circovirus type 2 (PCV2), with genome sizes of 1759 and 1767/1768 nucleotides, respectively [2]. PCV1 was first detected in 1974 as a contaminant of the porcine kidney cell line PK-15 (ATCC CCL-33) and was determined to be non-pathogenic [3]. However, PCV2 was found in pigs more than two decades later and proved to have the clinical manifestation of post-weaning multi-systemic wasting syndrome (PMWS) [4].

Globally, PCV2 is recognized as an emerging swine pathogen of great economic importance, causing huge losses in the piggery business. The viral pathogen has been the major culprit in cases of PMWS and other clinical disease manifestations in pigs that are generally regarded as porcine circovirus-associated diseases (PCVADs) [5]. These include porcine dermatitis and nephropathy syndrome (PDNS), porcine circovirus reproductive disorders, porcine respiratory disease complex, enteritis, acute pulmonary oedema, nervous system lesions, proliferative and necrotizing pneumonia, and a recently resurfacing neonatal congenital tremor [5, 6].

The viral genome of PCV2 is composed of at least five open reading frames (ORFs) that could be transcribed; however, ORF1 (coding for replication proteins) and ORF2 (coding for structural capsid protein) remain the widely sequenced and studied regions [7]. PCV2 generally infects 7–16-week old weaners and growers, as younger pigs are protected by passive immunity conferred by acquired maternal antibodies [8]. PCV2 infection normally affects the immune system of infected pigs by causing lymphoid tissue depletion, which leads to observable histological lesions. Co-infections with other pathogens normally result in worsened conditions in the infected pigs and is made possible due to the immunosuppression and reduced immunity that result from the PCV2 attacks on the protection system [5].

PCV2 strains have been classified into four main genotypes (PCV2a, PCV2b, PCV2c, and PCV2d) based on phylogenetic analyses performed with their full genomes and ORF2 sequences [9]. Previously, PCV2c was detected only in Denmark from archived materials; however, it was recently found in live feral pigs in Brazil and from field samples taken from sick pigs in China [10–12]. The duo of PCV2a and PCV2b has a worldwide distribution; since 2003 however, there has been an observable worldwide genotypic shift in occurrence from PCV2a to PCV2b in pig herds, which has since made PCV2b the predominant genotype [13]. PCV2d (previously regarded as a mutant PCV2b) is the newly emerging genotype that is currently circulating the globe in swine herds and has an apparent higher virulence [14, 15].

PCV2-infected pigs normally shed the virus through many routes, including urine, faeces, milk, oronasal secretions, colostrum, and semen [16, 17], thereby enhancing the transmission and spread of PCV2 infections among local herds. The global transmission of PCV2 infections is also greatly enhanced through the international trade of live pigs and pig products due to the subtle nature of the disease, which can be in the form of a subclinical infection [13, 18, 19]. Hence, proper diagnostic measures to check for the wild spread of the viral pathogen become imperative. There are two major ways of diagnosing PCV2 infections. Preliminarily, this can be done by observing for clinical signs, but this may not be accurate or reliable in cases of subclinical infections [20]. The confirmatory detection of PCV2 nucleic acids or antigens in samples from infected animals is therefore imperative. This has been achieved over the years through polymerase chain reaction (PCR), immunohistochemistry (IHC), and in situ hybridization techniques [5].

Although PCV2 is rampant in domestic pig populations globally, severe PCVAD may not occur on very many occasions. Not all pigs within the affected herd develop PCVAD because PCV2 is not the only factor required for disease expression [20]. As a multifactorial disease, there have been numerous studies on infectious and non-infectious cofactors in PCV2 infections, which include the co-infection of PCV2 and other pathogens [20]. Non-infectious cofactors of PCV2 infection include the genetic background of the pig, management practices such as high stocking density, and prevailing environmental conditions such as temperature fluctuations within the pen [21, 22].

Being a multifactorial disease, the prevention and control of PCVAD have been achieved over time through all-encompassing measures that consider both infectious and non-infectious contributing agents. Although vaccination has proven effective in preventing PCV2 infection and spread within the herds, the effectiveness is better achieved by combining it with good management practices such as preventing cross-fostering, strict biosecurity practice, careful breed selection, maintaining high standards of hygiene through the effective use of disinfectants, and good housing conditions [23, 24].

Despite the ubiquitous status of the virus, it has recently been revealed that the virus is grossly understudied in sub-Saharan Africa and on the African continent at large [25]. In South Africa, PCV2 was first detected on a commercial breeding farm in 2001 in pigs with clinical manifestations of PDNS and PMWS [26]. To date, however, no large-scale study has been conducted on the prevalence of PCV2 and its associated diseases to ascertain the true infection status of the pigs in that country. The recommendations by Drew et al. [26] and An et al. [27] for further molecular epidemiological studies on PCV2 strains at other premises in South Africa have been grossly neglected.

Therefore, the focus of this study was to validate the current PCV2 status of pigs in South Africa by surveilling for its presence in the swine herds of Eastern Cape Province. This serves as the first surveillance on the pathogen’s occurrence in the swine herds of this region and helps to contribute more South African-generated PCV2 sequences to GenBank, as there are currently very few from the country. In addition, it serves as motivation to conduct further studies in the near future entailing wider geographic regions to accurately document the virus genogroups circulating in the country and the entire sub-Saharan African region.

## Results

### Characteristics and observable farm management practices on the sampled farms

Three hundred and seventy-five blood, faecal, and nasal-swab samples were collected from seven commercial and communal pig farms in three district municipalities of Eastern Cape Province in the years 2015 and 2016. From the administered questionnaire, it was obvious that virtually all the managing personnel of the sampled farms were ignorant of PCV2 and its associated diseases. Moreover, the best practices that are required in a pig farming operation to prevent the transmission and outbreak of infectious diseases were essentially absent on most of the communal farms. Only one of the seven sampled farms employed high biosecurity measures in their piggery operations. It was also highly notable that none of the farms vaccinated their pigs against PCV2, not even the sampled commercial farm. More than half (57%) of the sampled farms were not using disinfectants for routine cleaning of their pens. Furthermore, a majority of the sampled farms had experienced some PCVAD symptoms such as wasting, abortion, and respiratory distress (Table 1).

**Table 1:** Farms features and some management practices of sampled farms

Farm code	Farm one (2FTP)	Farm two (TSO)	Farm three (MTH)	Farm four (CHA)	Farm five (CHB)	Farm six (CHC)	Farm seven (CHD)
Herds population	>5000	100	80	200	250	50	70
Herds composition	Weaners and growers	Sows, weaners, growers and boars	Sows, weaners, growers and boars	Sows, weaners, growers and boars	Sows, weaners, growers and boars	Sows, weaners, growers and boars	Sows, weaners, growers and boars
Breeds	Large white and Landrace	Large white	Large white	Large white and Landrace	Large white and Landrace	Large white	Large white
Biosecurity measures	High	Very low	None	Very low	Low	None	None
Biocide use	Virocide	Nil	Nil	Dazzel dip	Dazzel dip	Nil	Nil
Vaccination regime	Nil	Nil	Nil	Nil	Nil	Nil	Nil
Antibiotics use	Applied in cases of infections	Occasionally in cases of infections	Applied in cases of infections	Applied in cases of infections	Occasionally in cases of infections	Occasionally in cases of infections	Occasionally in cases of infections
Antibiotic/drugs use	Norotrim, Depomycin, Lantrax, Kyroligo, Advocin UltaJet,	Iron, Ivomec / dectomaxn	Iron, Ivomec / dectomax	Iron, Ivomec / dectomax	Iron, Ivomec / dectomax	Iron, Ivomec / dectomax	Iron, Ivomec / dectomax
All-in/All-out practice	Yes	No	No	Yes, but not always	Yes, but not always	No	No
Disease symptoms and occurrence	Swollen lymph nodes, meningitis, respiratory disorders, red cat, gastric ulcers, scrotal and navel hernia	Swollen lymph nodes, respiratory disorders	Meningitis, respiratory disorders, gastric ulcers, hernia	Wasting, Swollen lymph nodes, respiratory disorders, dysentery	Respiratory disorders	Swollen lymph nodes	Swollen lymph nodes, meningitis, respiratory disorders
Prior knowledge of PCV2 infections	Yes	No	No	No	No	No	No
Cases of abortion	Not applicable	Occasionally	Occasionally	Occasionally	Occasionally	Occasionally	Occasionally

### Molecular detection, characterization, and analysis of PCV2 DNA sequences from the field samples

Using PCV2-specific primers, the screened samples showed that the farm-level occurrence of PCV2 ranged from 5.6 to 60%, while overall, 54 of the 339 screened samples were positive for PCV2, representing 15.93% (Table 2). The assembly of the two amplified and sequenced PCV2 DNA fragments yielded nucleotide sequences of ~1041 nucleotides, which spanned a large portion of the replicase gene region of the viral genome and small portion of the structural (capsid) gene region.

**Table 2:** Farm level occurrence of PCV2

District Municipalities	Farms (Codes)	Farm types	Total Samples collected	Sampling Year	Samples screened	Positive samples	Positive samples (%)
AMATHOLE	2FTP	Commercial	206	2015	206	19	9.22
O.R. TAMBO	TSO	Communal	24	2015	24	2	8.33
	MTH	Communal	14	2015	14	2	14.29
CHRIS-HANI	CHA	Communal	35	2016	35	21	60.00
	CHB	Communal	59	2016	36	2	5.56
	CHC	Communal	13	2016	ND	ND	ND
	CHD	Communal	24	2016	24	8	33.33

*ND* Not Determined

**Fig. 1:**
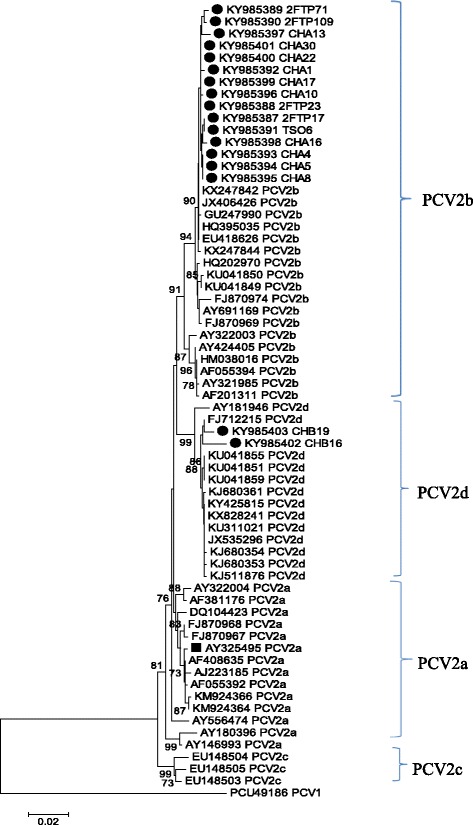
Phylogenetic analysis of the partial genomes of the PCV2 from this study with other sequences from GenBank. To further compare the derived sequences in this study with globally reported PCV2 sequences curated in GenBank, a phylogeny was performed using reference sequences from different geographic regions of the world in order to determine the evolutionary origins of the study sequences. The tree was constructed using 64 partial genomes of PCV2 (1041 nt) which comprised of 17 strains from this study and 47 other sequences obtained from GenBank while PCV1 sequence served as an out-group. The construction was done by using a Neighbor Joining algorithm and the percentage of replicate trees in which the associated taxa clustered together in the bootstrap test (1000 replicates) are shown next to the branches. The only PCV2 strain previously submitted to the GenBank from South Africa is indicated with the black square box. Analyses were conducted in MEGA6 [38]

**Fig. 2:**
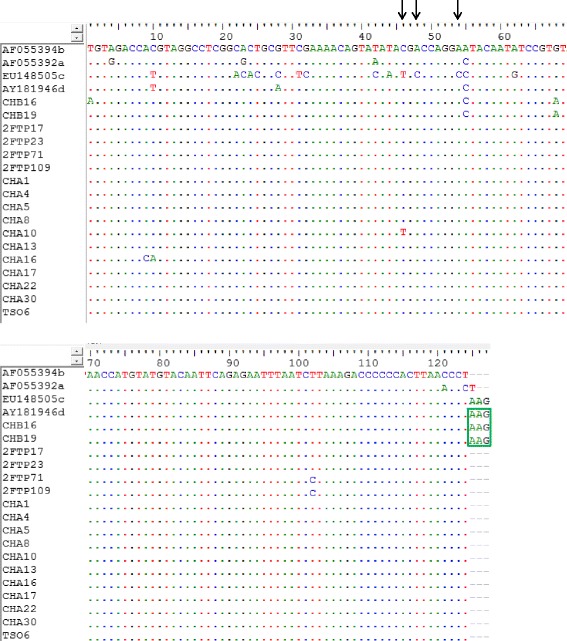
Nucleotide sequence alignment of a fragment of ORF2 genes of the 17 PCV2 strains. A segment of aligned PCV2 nucleotide sequences from this study and homologous reference sequences of four major genogroups of PCV2 obtained from GenBank. The green box shows the 3′ end of ORF2 gene where the stop codon mutation (TAA → AAG) gives rise to longer ORF2 gene (705 nt) that characterizes PCV2d capsid gene [39]. The black arrows show some of the marker nucleotides that differentiate PCV2c from PCV2d [9]

**Fig. 3:**
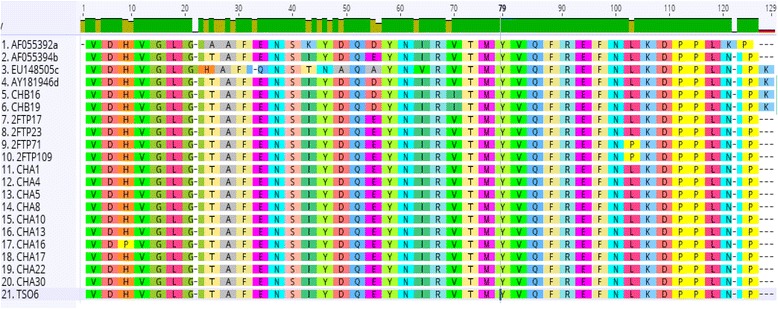
Amino acid sequence alignment of the partial capsid protein of the PCV2 strains. A segment showing alignment of the deduced amino acid (aa) sequences of the 17 PCV2 from this study and the reference sequences of four major genogroups of PCV2. Mutations at the C terminus of the capsid protein are shown, resulting to elongation of the ORF2 protein by one Lysine (K) residue in sample CHB16, CHB19 and PCV2d reference sequence (AY181946) as indicated by the green box compared to other 15 sequences in this study that formed the same pattern with reference PCV2b sequence (AF055394)

Using the Basic Local Alignment Search Tool (BLAST) analysis from the National Center for Biotechnology Information (NCBI) database, a homology search of the generated sequences confirmed that all 17 sequences were PCV2. Furthermore, a molecular analysis of the selected sequences revealed that 15 (88.2%) of the 17 PCV2-positive sequences clustered with other PCV2b sequences from different parts of the world, while the remaining two (11.8%) were grouped with the PCV2d reference sequences (Fig. 1). Both the nucleotide and amino acid sequence alignments of the PCV2 sequences from this study in comparison with the representative PCV2 reference sequences of the four major genogroups of importance in the global pig industry showed that the PCV2 sequences from this study belonged to the PCV2b and PCV2d genotypes (Figs. 2 and 3).

## Discussion

Infectious swine pathogens such as PCV2 have greatly attracted the attention of researchers since the early 1990s due to the economic havoc they produced in many pig-producing countries of the world [18]. Since 1997, PCV2 has been one of the most studied pig pathogens and has been referred to as an everlasting, worldwide, endemic pathogen in swine [28]. Since its first detection, there has been a notable successive change in the predominance of the circulating viral pathogen genogroup at the global level, changing from PCV2a before the year 2003 to PCV2b after that year [13]. Presently, PCV2d is becoming the most rampant and ravaging group with its observably higher virulence [15]. Despite the concern and global efforts to study the epidemiology of the viral pathogen, there has been a seemingly lethargic approach to its study in South Africa and the entire sub-Saharan African countries [25]. However, the scant information available has confirmed the presence of different PCV2 genogroups in the region, as the previously called North American-like strain [PCV2 group 2 (PCV2a)] was initially detected in South Africa in 2001 [26], and the European cluster [PCV2 group 1 (PCV2b)] was found in Ugandan pigs over a decade later [29]. Considering the economic importance of pig production in the developing countries of the world [30], the importance of this study in determining the PCV2 status of pigs on a relatively larger scale cannot be overemphasized.

In this study, 54 of the 339 samples screened for the presence of PCV2 were positive for the pathogen, confirming its presence on all of the tested farms. In addition, the farm level severity of occurrence was as high as 60% on one of the surveyed farms. This supports the ubiquitous status of PCV2 in swine herds globally [20]. Some selected high-quality amplicons were sequenced and processed to obtain 17 partial PCV2 genomes that were then analysed. The phylogenetic analyses confirmed that 15 of the PCV2 sequences formed a group together with the PCV2b reference sequences and were closely clustered with the Chinese strains NQY1 (KX247842), FX1102 (JX406426), FJMH0508 (GU247990), 09HuB (HQ395035), RUZHOU (EU418626), and XT1 (KX247844). The remaining two PCV2 sequences were grouped with the PCV2d GenBank sequences, being closest to the Xuancheng (FJ712215) strain from China. The only available reference sequence from South Africa, strain SA1 (AY325495), maintained its position among the other PCV2a reference strains used in this analysis, thus confirming the findings of Drew et al. [26] (Fig. 1).

The topography observed in the phylogenetic clustering was further confirmed through the nucleotide and amino acid sequence alignments (Figs. 2 and 3), in which CHB16 and CHB19 were found to have mutations in the stop codon at the 3′ end of ORF2, leading to an amino acid (Lysine-K) extension. This is a typical feature that distinguishes the mutant PCV2b (mPCV2b, now categorized as PCV2d), which is known to be more virulent than PCV2a and 2b [14]. This outcome agrees with the observed global PCV2 infection pattern, which has had a notable genotypic shift around the world, first from PCV2a to PCV2b, and recently to PCV2d [15, 31, 32]. The same incidence of genotypic shift may have also occurred in South African pigs since Drew et al. [26] first reported the case of a PCV2a outbreak in 2001, which was traced to inseminating the affected gilts with imported semen from Iowa, North America. This assertion was made due to the clustering of the first South African PCV2 strain (SA1) with many other sequences from there and Asia.

Alternatively, it is possible that the PCV2b and 2d strains detected in this study have been in circulation within the country all this time, but undetected. This claim might be substantiated on the basis that the initial detection in 2001 was from a case study of an outbreak in a commercial breeding unit that supplied breeding stock to other smaller farms [26] rather than from a large-scale surveillance study. In that case study, only 4 tissue samples from affected pigs were sent to the United Kingdom for analysis. Hence, an unnoticed transmission of different PCV2 genotypes from one herd to another could have been occurring within the country for many years prior to their being detected. Considering it was present at all of the tested farms, another scenario is that the PCV2b strains detected in this study entered through another route and are now spreading rapidly within the eastern region of the country. The occurrence of PCV2d in this study is highly significant and may represent the generally believed genotype shift from PCV2b to 2d, as has been observed in swine herds elsewhere in the world [15]. However, achieving a better understanding of this requires further prevalence studies and an in-depth molecular characterization of the PCV2 genotypes in circulation within the country.

The findings from this study suggest that the probable cost of neglecting proper PCV2 surveillance in South African pigs, as stated by Mokoele et al. [33], could be grievous and devastating with any instance of a large-scale PCV2 infection outbreak in the country. It is noteworthy that PCV2 was detected in all the sampled farms in the province, indicating a significant level of occurrence of the ‘small but powerful’ viral pathogen in the swine herds. The claim of negligence on the part of stakeholders in the country’s swine industry is further corroborated by the findings of our investigation. The pig farmers in the area had essentially no level of awareness (Table 1), and hence, were currently implementing no preventive measures.

Good management practice has been considered paramount in the prevention and control of PCV2 infections [24]. It is therefore not surprising that PCV2 was detected on all the sampled farms in this study, owning to the fact that the management practices of the majority of the farms was very poor. For example, a large percentage of the farms had little or no biosecurity measures in place that could forestall the farm-to-farm transmission of infectious agents such as PCV2. Furthermore, other important recommendations made in 1997 in Madec’s proposed 20-point plan to lower the impact of the disease [23] were grossly absent on the farms studied, such as the maintenance of good hygiene through the effective use of disinfectants and an all-in/all-out stocking practice. It is noteworthy, however, that despite the observance of good management practices, PCV2 was still present at the commercial farm (farm one) in the study. This observation further stresses the importance of an effective vaccination regime against PCV2 as part of an all-encompassing strategy in preventing infection [23, 24].

As a multifactorial disease having both infectious and non-infectious co-factors, PCVAD prevention and control rely on a prompt and adequate diagnosis, which is a function of efficient surveillance to ascertain the status of circulating PCV2 genotypes in the swine population. This normally informs the adoption of an effective vaccination regime and good management procedures to forestall rapid spread and the incidence of large-scale outbreaks. According to Segalés [34], continuous surveillance for new PCV2 variants is important for a DNA virus that, as Firth et al. report, has evolutionary rates comparable to that of RNA viruses [35]. The observable gross negligence by the stakeholders regarding PCV2 infection in South Africa and sub-Saharan African countries [25] at large could suppress the huge potential suggested for pig production to alleviate poverty and help solve hunger issues in developing countries [30].

## Conclusion

The detection of PCV2b and its mutant strain (PCV2d) in the swine herds of Eastern Cape Province after first detecting PCV2a in South African pigs more than a decade and half ago is very disturbing considering the economic importance of this viral pathogen. It is therefore extremely important that a rigorous enlightenment of the farmers about PCV2 and its associated diseases be undertaken in the country. This is highly imperative, as our survey revealed that virtually all of the farmers in the region were ignorant of the pathogen. An effective large-scale vaccination regime should also be initiated to curtail the rapid circulation of the virus and prevent impending future outbreaks. This should be done pari passu with educating the farmers (especially the communal ones) on the biosecurity measures required in piggery operations for optimal performance. Further studies on the prevalence of PCV2 and its co-infecting pathogens in the pigs of Eastern Cape Province and other South African provinces should also be conducted without further delay, with a candid effort geared towards obtaining more of the viral genomes (preferably complete) in circulation within the country. This will provide a thorough and immediate molecular characterization of the circulating strains and better our understanding of the epidemiology of this viral pathogen, both within the country and beyond.

## Methods

### Sample collection

A total number of three hundred and seventy five (375) field samples, consisting of blood, faecal and nasal swabs samples were collected from seven (commercial, semi-commercial and communal) farms from three District Municipalities of Eastern Cape Province, South Africa in 2015 and 2016. The fresh samples were randomly collected from both healthy and diseased pigs of the sampled farms; they were processed and kept in −80 °C freezer until when used. A Questionnaire was also designed (Additional file 1) and administered to each of the sampled farm to obtain information about some farm management practices and level of awareness on PCV2 from the farm managers.

### DNA extraction and PCV2 detection

Extraction of total genomic DNA was done from the processed samples by using ReliaPrep™ gDNA Tissue Miniprep System (Promega, Madison, USA) with strict adherence to the manufacturer procedures. Initial screening of the samples was performed through polymerase chain reaction (PCR) by using the primer pair P1 and P2, and subsequently, positive samples were again subjected to a second round of PCR amplification by using a primer pair P3 and P4 (Table 3). The first primer pair amplified a chunk of about 629 base pair (bp) long of ORF1 region (replicase gene) of the viral genome while the second overlapping primer pair amplified the remaining part of the ORF1 region and a portion of ORF2 (capsid gene) totaling about 630 bp in length.

**Table 3:** Primer pairs used for conventional PCR screening and subsequent nucleotide sequencing of partial PCV2 genomes

Primer identity	Primer sequence	Amplicon length	Nucleotide Position	Reference
P1Fw	5′-TAATCCTTCCGAAGACGAGC-3′	629	116–135	[27]
P2Rv	5′-CGATCACACAGTCTCAGTAG-3′	629	726–745	[27]
P3Fw	5′-CAGAAGCGTGATTGGAAGAC-3′	630	531–550	[27]
P4Rv	5′-ATGTAGACCACGTAGGCCTC-3′	630	1142–1161	[27]

The PCR amplification for PCV2 detection was carried out as earlier described by An et al. [27] with some modifications. The PCR reaction mixtures were made by adding 5 μL of extracted DNA to 45 μL of a reaction mixture containing a final concentration of 1.25 mM MgCl_2_, 5X PCR buffer, 0.2 mM dNTPs, 10 pmol of each primer, and 2.5 U of Taq DNA polymerase. PCR amplification was done in a MyCycler™ (Thermer Cycler 1.065) machine (Bio-Rad, Apllied Biosytem, California) with amplification conditions of 95 °C for 4mins (initial denaturation); 35 cycles (final denaturation of 95 °C for 30 s, annealing of 57 °C for 30 s, elongation of 72 °C for 1 min); final elongation of 72 °C for 5mins. PCR products were analyzed by electrophoresis on 1.5% Agarose gels stained with Ethidium bromide (EB) and visualized using an Alliance 4.7 transilluminator (UVitec, Cambridge, UK).

### Sequencing of amplified nucleotide sequences, analysis and construction of phylogenetic tree

Positive PCR products of high quality were selected for sequencing at University of Stellenbosch Central DNA Sequencing Facility using the forward and reverse primers earlier used in PCR amplification. Post PCR clean-ups were performed using Nucleofast 96 well PCR plate (Macherey-Nagel, Düren, Germany) with adherence to the manufacturer’s instructions on a Tecan EVO150 robotic workstation (Tecan Group, Männedorf, Switzerland). The purified products were sequenced with standard Sanger sequencing using the BigDye Terminator V3.1 sequencing kit (Applied Biosystems, Foster City, CA, USA) in line with the manufacturer’s instructions with slight modifications. Sequenced DNA were edited, blasted and assembled using Geneious 10.1.2 [36].

The execution of Basic Local Alignment Search Tool (BLAST) was carried out on the DNA sequences as an initial measure to ascertain that all the sequences were truly PCV2 in comparison with other sequences present in the GenBank. Nucleotide sequence alignment was performed by using Bioedit software [37] while amino acid alignment was done by using ClustalW as implemented in Geneious 10.1.2 software [36]. The Phylogenetic tree was reconstructed using the distance-based neighbor joining algorithm as implemented in Mega 6 [38]. Reliability was evaluated by the bootstrapping method on 1000 replicate of the alignment. All sequences that were obtained from GenBank and used for reconstructing the phylogenetic tree are as follow: PCU49186, AY556474, AY325495, AY322004, AJ223185, AF408635, AF381176, KM924366, KM924364, FJ870968, FJ870967, DQ104423, AF055392, AY691169, AY424405, AY322003, AY321985, KU041850, KU041849, HQ202970, HM038016, FJ870974, FJ870969, AF055394, KX247842, JX406426, EU418626, HQ395035, GU247990, KX247844, AF201311, EU148503, EU148504, EU148505, KU041859, KU041855, KU041851, KJ680361, KJ680354, KJ680353, KJ511876, KX828241, AY181946, FJ712215, JX535296, KY425815 and KU311021 (see Additional file 2 for details).

## Additional files

Additional file 1: Questionnaire on porcine circovirus type 2 (PCV2) study**.** Designed questionnaire that was used to obtain information about some farm management practices and level of awareness on PCV2 from the managers of the sampled farms. (DOCX 17 kb)

Additional file 2: PCV2 sequences in this study and other reference sequences reported previously that were used in the phylogenetic analysis. Details of PCV2 sequences from this study and other reference sequences obtained from GenBank. (PDF 89 kb)

## Abbreviations

BLAST: Basic local alignment search tool
DNA: Deoxyribonucleic acid
IHC: Immunohistochemistry
NCBI: National Center for Biotechnology Information
ORF: Open reading frame
PCR: Polymerase chain reaction
PCVAD: Porcine circovirus associated disease
PCVs: Porcine circoviruses
PDNS: Porcine dermatitis and nephropathy syndrome
PMWS: Postweaning multisystemic wasting syndrome

## References

[1] Tischer I, Gelderblom H, Vettermann W, Koch MA (1982). A very small porcine virus with circular single-stranded DNA. Nature.

[2] Meehan BM, McNeilly F, Todd D, Kennedy S, Jewhurst VA, Ellis JA, Hassard LE, Clark EG, Haines DM, Allan GM (1998). Characterization of novel circovirus DNAs associated with wasting syndromes in pigs. J Gen Virol.

[3] Tischer I, Rasch R, Tochtermann G (1974). Characterization of papovavirus-and picornavirus-like particles in permanent pig kidney cell lines. Zentralbl Bakteriol Orig A.

[4] Allan GM, Ellis JA (2000). Porcine circoviruses: a review. J Vet Diagn Investig.

[5] Opriessnig T, Meng XJ, Halbur PG (2007). Porcine circovirus type 2–associated disease: update on current terminology, clinical manifestations, pathogenesis, diagnosis, and intervention strategies. J Vet Diagn Investig.

[6] Tummaruk P, Pearodwong P (2016). Porcine circovirus type 2 expression in the brain of neonatal piglets with congenital tremor. Comp Clinical Patholog.

[7] Franzo G, Tucciarone CM, Cecchinato M, Drigo M (2016). Porcine circovirus type 2 (PCV2) evolution before and after the vaccination introduction: a large scale epidemiological study. Sci Rep.

[8] McKeown NE, Opriessnig T, Thomas P, Guenette DK, Elvinger F, Fenaux M, Halbur PG, Meng XJ (2005). Clinic Vacc Immunol.

[9] Franzo G, Cortey M, Olvera A, Novose D, De Castro AM, Biagini P, Segalés J, Drigo M (2015). Revisiting the taxonomical classification of porcine Circovirus type 2 (PCV2): still a real challenge. Virol J.

[10] Dupont K, Nielsen EO, Bækbo P, Larsen LE (2008). Genomic analysis of PCV2 isolates from Danish archives and a currentPMWS case-control study supports a shift in genotypes with time. Vet Microbiol.

[11] Franzo G, Cortey M, de Castro AM, Piovezan U, Szabo MP, Drigo M, Segalés J, Richtzenhain LJ (2015). Genetic characterisation of porcine circovirus type 2 (PCV2) strains from feral pigs in the Brazilian Pantanal: an opportunity to reconstruct the history of PCV2 evolution. Vet Microbiol.

[12] Liu X, Wang F, Zhu H, Sun N, Phylogenetic WH (2016). Analysis of porcine circovirus type 2 (PCV2) isolates from China with high homology to PCV2c. Arch Virol.

[13] Franzo G, Cortey M, Segalés J, Hughes J, Drigo M (2016). Phylodynamic analysis of porcine circovirus type 2 reveals global waves of emerging genotypes and the circulation of recombinant forms. Mol Phylogenet Evol.

[14] Guo L, Fu Y, Wang Y, Lu Y, Wei Y (2012). A porcine circovirus type 2 (PCV2) mutant with 234 amino acids in capsid protein showed more virulence in vivo, compared with classical PCV2a/b strain. PLoS One.

[15] Xiao CT, Halbur PG, Opriessnig T (2015). Global molecular genetic analysis of porcine circovirus type 2 (PCV2) sequences confirms the presence of four main PCV2 genotypes and reveals a rapid increase of PCV2d. J Gen Virol.

[16] Patterson AR, Madson DM, Halbur PG, Opriessnig T (2011). Shedding and infection dynamics of porcine circovirus type 2 (PCV2) after natural exposure. Vet Microbiol.

[17] Segalés J, Calsamiglia M, Olvera A, Sibila M, Badiella L, Domingo M (2005). Quantification of porcine circovirus type 2 (PCV2) DNA in serum and tonsillar, nasal, tracheo-bronchial, urinary and faecal swabs of pigs with and without postweaning multisystemic wasting syndrome (PMWS). Vet Microbiol.

[18] Vidigal PM, Mafra CL, Silva FM, Fietto JL, Silva Junior A, Almeida MR (2012). Tripping over emerging pathogens around the world: a phylogeographical approach for determining the epidemiology of porcine circovirus-2 (PCV2), considering global trading. Virus Res.

[19] Franzo G, Tucciarone CM, Dotto G, Gigli A, Ceglie L, Drigo M (2015). International trades, local spread and viral evolution: the case of porcine circovirus type 2 (PCV2) strains heterogeneity in Italy. Inf Gen Evol.

[20] Gillespie J, Opriessnig T, Meng XJ, Pelzer K, Buechner-Maxwell V (2009). Porcine circovirus type 2 and porcine circovirus-associated disease. J Vet Intern Med.

[21] Li Y, Liu H, Wang P, Wang L, Sun Y, Liu G (2016). RNA-Seq analysis reveals genes underlying different disease responses to porcine circovirus type 2 in pigs. PLoS One.

[22] Patterson R, Nevel A, Diaz AV, Martineau HM, Demmers T, Browne C, Mavrommatis B, Werling D (2015). Exposure to environmental stressors result in increased viral load and further reduction of production parameters in pigs experimentally infected with PCV2b. Vet Microbiol.

[23] Madec F, Rose N, Eveno E, Morvan P, Larour G, Jolly JP, Le Diguerher G, Cariolet R, Le Dimna M, Blanchard P, Jestin A. PMWS: on-farm observations and preliminary analytic epidemiology. In: proceedings of ssDNA viruses of plants, birds, pigs and primates, Saint-Malo, France, 24–27 September 2001. 2001;86–87.

[24] Rose N, Larour G, Le Diguerher G, Eveno E, Jolly JP, Blanchard P, Oger A, Le Dimna M, Jestin A, Madec F (2003). Risk factors for porcine post-weaning multisystemic wasting syndrome (PMWS) in 149 French farrow-to-finish herds. Prev Vet Med.

[25] Afolabi KO, Iweriebor BC, Okoh AI, Obi LC (2017). Global status of *Porcine circovirus 2* and its associated diseases in sub-Saharan Africa. Adv Virol.

[26] Drew TW, Grierson SS, King DP, Hicks D, Done S, Neser JA, Evans DP, Grimbeek P, Banks M (2004). Genetic similarity between porcine circovirus type 2 isolated from the first reported case of PMWS in South Africa and north American isolates. Vet Rec.

[27] An DJ, Roh IS, Song DS, Park CK, Park BK (2007). Phylogenetic characterization of porcine circovirus type 2 in PMWS and PDNS Korean pigs between 1999 and 2006. Virus Res.

[28] Segalés J, Kekarainen T, Cortey M (2013). The natural history of porcine circovirus type 2: from an inoffensive virus to a devastating swine disease?. Vet Microbiol.

[29] Ojok L, Okuni JB, Hohloch C, Hecht W, Reinacher M (2013). Detection and characterisation of porcine circovirus 2 from Ugandan pigs. Indian J. Vet Pathol.

[30] FAO. Pig Sector Kenya. FAO animal production and health livestock country reviews. Rome 2012;3.

[31] Cortey M, Pileri E, Sibila M, Pujols J, Balasch M, Plana J (2011). Genotypic shift of porcine circovirus type 2 from PCV-2a to PCV-2b in Spain from 1985 to 2008. Vet J.

[32] Huang L, Wang Y, Wei Y, Chen D (2016). Capsid proteins from PCV2a genotype confer greater protection against a PCV2b strain than those from PCV2b genotype in pigs: evidence for PCV2b strains becoming more predominant than PCV2a strains from 2000 to 2010. App. Microbiol Biotechnol.

[33] Mokoele JM, Janse van Rensburg L, vanLochem S (2015). Overview of the perceived risk of transboundary pig diseases in South Africa. J South Afric Vet Assoc.

[34] Segalés J (2015). Best practice and future challenges for vaccination against porcine circovirus type 2. Expert Rev Vacc.

[35] Firth C, Charleston MA, Duffy S (2009). Insights into the evolutionary history of an emerging livestock pathogen: porcine circovirus 2. J Virol.

[36] Kearse M, Moir R, Wilson A, Stones-Havas S (2012). Geneious basic: an integrated and extendable desktop software platform for the organization and analysis of sequence data. Bioinformatics.

[37] Hall T (1999). BioEdit: a user-friendly biological sequence alignment editor and analysis program for windows 95/98/NT. Nucleic Acids Symp Ser.

[38] Tamura K, Stecher G, Peterson D, Filipski A, Kumar S (2013). MEGA6: molecular evolutionary genetics analysis version 6.0. Mol Biol Evol.

[39] Guo LJ, Lu YH, Wei YW, Huang LP, Liu CM (2010). Porcine circovirus type 2 (PCV2): genetic variation and newly emerging genotypes in China. Virol J.

