# Supplementary material for: Molecular detection of Porcine circovirus type 2 in swine herds of Eastern Cape Province South Africa
Source: BMC Microbiol. 2017 Nov 2;17:212. doi: 10.1186/s12866-017-1121-4 (PMC5669008; doi:10.1186/s12866-017-1121-4)
Supplement: Supplementary file 1 — Questionnaire on porcine circovirus type 2 (PCV2) study. Designed questionnaire that was used to obtain information about some farm management practices and level of awareness on PCV2 from the managers of the sampled farms. (DOCX 17 kb) [file 12866_2017_1121_MOESM1_ESM.docx]

| **Additional file 1: PCV2 sequences in this study and other reference sequences reported previously that were used in the phylogenetic analysis.** | | | |
| --- | --- | --- | --- |
| **Genotype** | **Name** | **Geographical location** | **Source** |
| **PCV1** | PCU49186 | Northern Ireland UK | GenBank |
| **PCV2a** | AY556474 | China | GenBank |
|  | AY325495 | South Africa | GenBank |
|  | AY322004 | France | GenBank |
|  | AJ223185 | USA | GenBank |
|  | AF408635 | Canada | GenBank |
|  | AF381176 | China | GenBank |
|  | KM924366 | South Korea | GenBank |
|  | KM924364 | South Korea | GenBank |
|  | FJ870968 | China | GenBank |
|  | FJ870967 | China | GenBank |
|  | DQ104423 | China | GenBank |
|  | AF055392 | Canada | GenBank |
| **PCV2b** | AY691169 | China | GenBank |
|  | AY424405 | Austria | GenBank |
|  | AY322003 | France | GenBank |
|  | AY321985 | France | GenBank |
|  | KU041850 | China | GenBank |
|  | KU041849 | China | GenBank |
|  | HQ202970 | Taiwan | GenBank |
|  | HM038016 | China | GenBank |
|  | FJ870974 | China | GenBank |
|  | FJ870969 | China | GenBank |
|  | AF055394 | France | GenBank |
|  | KX247842 | China | GenBank |
|  | JX406426 | China | GenBank |
|  | EU418626 | China | GenBank |
|  | HQ395035 | China | GenBank |
|  | GU247990 | China | GenBank |
|  | KX247844 | China | GenBank |
|  | AF201311 | Germany | GenBank |
|  | KY985387 (2FTP17; Strain AFOS1-FH1) | South Africa | This study |
|  | KY985388 (2FTP23; Strain BEN1-FH2) | South Africa | This study |
|  | KY985389 (2FTP71; Strain OBI1-FH3) | South Africa | This study |
|  | KY985390 (2FTP109; Strain OKOH1-FH4) | South Africa | This study |
|  | KY985391 (TSO6; Strain AFOS2-TSO) | South Africa | This study |
|  | KY985392 (CHA1; Strain AFOS3-CHA1) | South Africa | This study |
|  | KY985393 (CHA4; Strain AFOS4-CHA2) | South Africa | This study |
|  | KY985394 (CHA5; Strain BEN2-CHA3) | South Africa | This study |
|  | KY985395 (CHA8; Strain BEN3-CHA4) | South Africa | This study |
|  | KY985396 (CHA10; Strain BEN4-CHA5) | South Africa | This study |
|  | KY985397 (CHA13; Strain OBI2-CHA6) | South Africa | This study |
|  | KY985398 (CHA16; Strain OBI3-CHA7) | South Africa | This study |
|  | KY985399 (CHA17; Strain OBI4-CHA8) | South Africa | This study |
|  | KY985400 (CHA22; Strain OKOH2-CHA9) | South Africa | This study |
|  | KY985401 (CHA30; Strain OKOH3-CHA10) | South Africa | This study |
| **PCV2c** | EU148505 | Denmark | GenBank |
|  | EU148504  EU148503 | Denmark  Denmark | GenBank  GenBank |
| **PCV2d** | KU041859 | China | GenBank |
|  | KU041855 | China | GenBank |
|  | KU041851 | China | GenBank |
|  | KJ680361 | China | GenBank |
|  | KJ680354 | China | GenBank |
|  | KJ680353 | China | GenBank |
|  | KJ511876 | China | GenBank |
|  | KX828241 | South Korea | GenBank |
|  | AY181946 | China | GenBank |
|  | FJ712215 | China | GenBank |
|  | JX535296 | USA | GenBank |
|  | KY425815 | China | GenBank |
|  | KU311021 | China | GenBank |
|  | KY985402 (CHB16; Strain AFOS5-CHB1) | South Africa | This study |
|  | KY985403 (CHB19; Strain AFOS6-CHB2) | South Africa | This study |
