# Supplementary material for: Molecular detection of Porcine circovirus type 2 in swine herds of Eastern Cape Province South Africa
Source: BMC Microbiol. 2017 Nov 2;17:212. doi: 10.1186/s12866-017-1121-4 (PMC5669008; doi:10.1186/s12866-017-1121-4)
Supplement: Supplementary file 2 — PCV2 sequences in this study and other reference sequences reported previously that were used in the phylogenetic analysis. Details of PCV2 sequences from this study and other reference sequences obtained from GenBank. (PDF 89 kb) [file 12866_2017_1121_MOESM2_ESM.pdf]

University of Fort Hare
Together in Excellence

QUESTIONNAIRE ON PORCINE CIRCOVIRUS TYPE 2 (PCV2) STUDY

1) Location of farm (District municipality):……………………………………….…………………………….

……………………………………………………………………………………………………...

2) Size of herd: ≤100 [ ] 101 – 1000 [ ] 1000 – 2000 [ ]

5000 and above [ ]

3) Ages of pigs: piglets [ ] growers [ ] finisher [ ] boars [ ]

Sows [ ]

4) What breed of pigs do you have in your farm? Large white [ ] Land race [ ]

Duroc [ ] Yorkshire [ ] Others…………………………………………………………….

……………………………………………………………………………………………………...

5) Observed sanitary/biosecurity measures: none [ ] low [ ] medium [ ]

High [ ] very high [ ]

6) What biocide do you use in the farm?..

……………………………………………………………………………………………………...

7) Do you vaccinate your animals? If so against which diseases?..

……………………………………………………………………………………………………...

……………………………………………………………………………………………………...

8) Types of antibiotics/drugs used and when?…………………………………………………………...

………………………………………………………………………………………………………...

9) Do you practice all-in/all-out pig rearing style in your farm? Yes [ ] No [ ]

10) Do you have veterinary personnel that attend to your animals? Yes [ ] No [ ]

11) How often does he/she visits your farm?...

12) What disease outbreaks have you ever experienced on your farm?..

………………………………………………………………………………………………………...

………………………………………………………………………………………………………...

13) How often do your pregnant sows have abortion?...

………………………………………………………………………………………………………

14) If you experience abortion among your sows what do you do? Sell the sow, treat it and leave it still in the farm?..

15) Breeding procedure Artificial insemination [ ] Natural crossing [ ]

16) Do you have a sick bay for diseased animals on your farm? Yes [ ] No [ ]

17) Have you heard of PCV2/post weaning multisystemic wasting syndrome? Yes [ ] No [ ]

18) If yes, have you noticed it among your piglets [ ] growers [ ] or finishers [ ]?

19) Observable clinical features of PCV2 associated diseases……………………………………………

……………………………………………………………………………………………………….

20) What kind of feeds do you use in your farm and where do you usually purchase it?..

....
